# Ab initio identification of human microRNAs based on structure motifs

**DOI:** 10.1186/1471-2105-8-478

**Published:** 2007-12-18

**Authors:** Markus Brameier, Carsten Wiuf

**Affiliations:** 1 Bioinformatics Research Center (BiRC), University of Aarhus, 8000 Aarhus C, Denmark

## Abstract

**Background:**

MicroRNAs (miRNAs) are short, non-coding RNA molecules that are directly involved in post-transcriptional regulation of gene expression. The mature miRNA sequence binds to more or less specific target sites on the mRNA. Both their small size and sequence specificity make the detection of completely new miRNAs a challenging task. This cannot be based on sequence information alone, but requires structure information about the miRNA precursor. Unlike comparative genomics approaches, *ab initio *approaches are able to discover species-specific miRNAs without known sequence homology.

**Results:**

MiRPred is a novel method for *ab initio *prediction of miRNAs by genome scanning that only relies on (predicted) secondary structure to distinguish miRNA precursors from other similar-sized segments of the human genome. We apply a machine learning technique, called linear genetic programming, to develop special classifier programs which include multiple regular expressions (motifs) matched against the secondary structure sequence. Special attention is paid to scanning issues. The classifiers are trained on fixed-length sequences as these occur when shifting a window in regular steps over a genome region. Various statistical and empirical evidence is collected to validate the correctness of and increase confidence in the predicted structures. Among other things, we propose a new criterion to select miRNA candidates with a higher stability of folding that is based on the number of matching windows around their genome location. An ensemble of 16 motif-based classifiers achieves 99.9 percent specificity with sensitivity remaining on an acceptable high level when requiring all classifiers to agree on a positive decision. A low false positive rate is considered more important than a low false negative rate, when searching larger genome regions for unknown miRNAs. 117 new miRNAs have been predicted close to known miRNAs on human chromosome 19. All candidate structures match the free energy distribution of miRNA precursors which is significantly shifted towards lower free energies. We employed a human EST library and found that around 75 percent of the candidate sequences are likely to be transcribed, with around 35 percent located in introns.

**Conclusion:**

Our motif finding method is at least competitive to state-of-the-art feature-based methods for *ab initio *miRNA discovery. In doing so, it requires less previous knowledge about miRNA precursor structures while programs and motifs allow a more straightforward interpretation and extraction of the acquired knowledge.

## Background

With the discovery of miRNAs the traditional view of RNA as pure helper molecules in protein biogenesis has changed radically. MiRNAs belong to a class of single-stranded, non-coding RNA (ncRNA) with only 21–25 nt in sequence length. They are directly involved in downregulation of gene expression at the post-transcriptional level, i.e., they act as negative regulators of translation, in multi-cellular animals and plants, and also appear in viruses (see [1-3] for reviews).

According to the current understanding of miRNA biogenesis, the primary miRNA (pri-miRNA) transcript is cleaved into a 60–70 nt long precursor sequence by the Drosha/Pasha complex. The pre-miRNA is transported into the cytoplasm by Exportin 5 and cleaved by Dicer into the mature miRNA. In the RISC (RNA-induced silencing complex) these molecules regulate expression of target genes by binding to complementary sites on the messenger RNA (mRNA). This causes either cleavage and degradation of the mRNA or just suppresses its translation into a protein.

**Figure 1:**
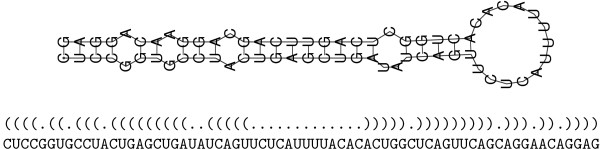
Typical hairpin structure and corresponding secondary structure sequence of miRNA precursor as predicted by RNAfold [22]. Base pairings are represented by complementary parentheses and non-paring bases by dots. Human miRNA mir-24-1 is shown.

The miRNA precursor sequence folds into the typical hairpin stem-loop structure (see Figure 1) which is considered to be the most important indicator in the maturation process. RNA folding is determined mainly by hydrogen bonds between complementary base pairs (C-G and A-U), and the wobble pair G-U. Basically, RNAs form *loops *or *bulges *by unpaired bases and continuous segments of base pairings (*stems*) as secondary structure components.

The structure of RNA molecules is key to their type and function. Simpler computational approaches to discover miRNA precursors in human or animals strongly rely on sequence homology to known miRNAs [4] or on sequence profiles [5]. Other algorithms have been based on characteristic secondary structure features and/or evolutionary conservation among different species [6-9]. Wang *et al. *[10] align both the secondary structure and the sequence of known miRNA precursors. Some methods consider the genomic context of miRNA precursors by using conservation patterns of the flanking sequences [11] or their appearance in clusters [12] as additional search criteria. Berezikov *et al. *[13] use phylogenetic shadowing to derive a general conservation profile over miRNA precursor and flanking sequences. This profile is used in combination with a structure filter to search for novel miRNAs.

All these methods make use of comparative genomics information. In contrast, *ab initio *prediction methods are able to predict miRNAs in a single genome without using comparative sequence analysis or requiring sequence or structure conservation. This enables the identification of completely new miRNAs for which no close homologs are known. It is estimated that the number of non-conserved miRNAs may be relatively large [14].

In [15] a support vector machine (SVM) is trained on frequencies of predefined structure-sequence triplets. The authors found a significantly higher specificity than when using counts of structure triplets only. Another feature-based SVM approach [16] uses nucleotide and base pair frequencies as input features in addition to lengths and distances of certain miRNA structure elements. Nam *et al. *[17] combine sequential and structural miRNA characteristics into a Hidden Markov Model (HMM) to identify distant homologs. Helvik *et al. *[18] employ two SVM classifiers trained on both sequence and structure features. The first-level SVM predicts Drosha processing sites which are used by the second-level SVM for an improved prediction of miRNA genes.

Here we introduce an *ab initio *method that relies *only *on characteristic patterns found or not found in the predicted secondary structure of miRNA precursors (see Figure 1), while ignoring the nucleotide sequence completely. Terminal loop and flanking sequences of the precursor occur to be more variable (less conserved) than the stem sequences [12,13], probably because of a minor importance during maturation. The mature sequence in the stem, however, is rather short and relatively specific to the binding sites on the target mRNA and, thus, to miRNA function. This leaves the question, to what extent characteristic sequence patterns may be shared by currently known and unknown miRNAs. We don't claim that sequence information can not be useful for *de novo *miRNA finding, but demonstrate what is possible without. 

We apply linear genetic programming (GP) [19] to develop classifier programs. Each program incorporates and combines multiple regular expressions (REs) that are matched against the input sequence. Here REs represent powerful *structure motifs *which may reveal new knowledge about miRNA precursors. Another advantage is that the method makes almost no assumptions about miRNAs, i.e., it does not require previous knowledge about concrete structural features besides the training data. In particular, the patterns searched for do not have to be predefined, but are adapted automatically (*de novo *motif finding). Because motif matching is position-independent, there is also no need to preselect candidate hairpins. The overall method for finding miRNA structures by scanning genome regions, named miRPred, may be summarized in five steps:

(1) A fixed-length window is shifted in regular steps over the input sequence and its secondary structure sequence is predicted.

(2) Non-miRNA structures are filtered out based on free energy and number of base pairings (optional).

(3) The core method applies several classifier programs to a windowed structure sequence and combines their predictions by voting with threshold.

(4) Double matches by overlapping sequence windows are detected and removed.

(5) The predicted miRNA candidates are filtered using a stricter threshold, e.g., on the number of successive matches around their genome location (optional).

The general motivation to combine multiple predictions is to increase confidence. Here an ensemble of 16 classifiers was trained on known miRNAs in the human genome. This number was necessary to achieve a specificity of 99.9 percent if all classifiers have to agree on a positive decision of the ensemble. A low false positive rate is preferable to a low false negative rate, when scanning wider genome regions for new miRNAs. A higher proportion of candidate hairpins, instead, might come along with too many false positive predictions.

## Results and discussion

### Data preparation

Machine learning methods derive classification models or rules from known positive and negative examples. Our predictors were trained and are applied on *fixed-length *example sequences (see Methods).

The positive set is composed of all 474 human miRNA hairpin sequences reported in miRBase (release 9.0) [20,21]. All miRNAs in miRBase are either verified experimentally or by comparative analysis, i.e., have easily identifiable homologs in other closely-related species. A 100 nt wide sequence window is centered and extracted around each known miRNA hairpin in the human genome (Ensembl NCBI36 release 42). That is, smaller hairpins are extended, while longer hairpins are cut equally at both ends. Note that many stem-loop sequences reported in miRBase are longer than the actual precursor (~70 nt) and contain flanking material from the primary transcript. The reverse complementary sequence is used if a miRNA is located on the antisense strand.

For the negative examples, we first select 20,000 random locations in the human genome. At each position we shift a 100 nt wide window 5 times in sense direction – using a constant step size of 5 nt – and extract the subsequence of each window. The resulting 100,000 fixed-length and partly overlapping sequences simulate the input situation for the predictors when applied for scanning genome regions. A higher number of negative samples is needed because of the higher diversity of non-miRNA structures. Moreover, it is relatively unlikely to include a significant proportion of real miRNAs in this way, also because of their assumed low frequency in the genome.

**Figure 2:**
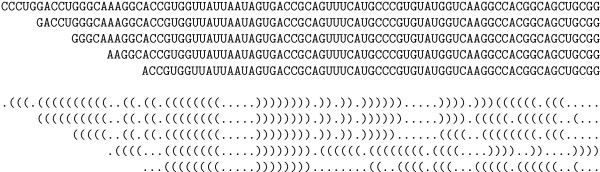
Example of shifted sequence window and corresponding secondary structure sequences. Some substructures are more stable than others.

The RNA secondary structure (stem-loop sequence) of all extracted nucleotide sequences is predicted using RNAfold (Vienna package release 1.4 [22,23]) at default temperature 37°C and with default settings. Figure 2 gives an example of 5 successive sequence windows during scanning and their corresponding stem-loop structures which are strings of base pairings (represented by complementary parentheses "(" and ")") and non-paring bases (dots "."). It is seen that some substructures are more stable, i.e., independent of the window position, than others.

### Prefiltering of stem-loop structures

Both data sets are prefiltered to rule out structures which are quite likely non-miRNAs. A candidate structure must meet three relatively weak conditions: (1) minimum 18 base pairings in the stem arm of the hairpin structure including the wobble pair G-U; (2) maximum -15 kcal/mol free energy. These thresholds derive from the lowest number of base pairing and the highest free energy found among known human pre-miRNAs. (3) Both conditions must be met by the structure of the central 70 nt sequence in the scanning window (100 nt). This is approximately the size and location of the miRNA precursor, ignoring the 15 nt extensions from the primary sequence. In doing so, only 5 (~1 percent) of the positive examples (human miRNAs) but 79,097 (~80 percent) negative examples are excluded.

Note that prefiltering is not essential for the application of our motif-based GP classifiers. Actually, we found that it affects the final set of predicted candidates only slightly. That is, the classifiers have learned to reject the prefiltered structures even though they have not been exposed to such during training. Nevertheless, prefiltering is important for a reduction and better selection of the training examples. 

Different arguments may be found for using a scanning window of 100 nt: (1) The ~70 nt long pre-miRNA sequence may not always be perfectly aligned in the window center. (2) Short single-stranded extensions from the primary transcript flanking both ends of the precursor are argued to carry relevant structural information for Drosha processing [24,25]. The secondary structure of a 13 nt region following the mature duplex has been shown to be conserved [26]. (3) Larger windows may include too much of the pri-miRNA structure. (4) Folding of miRNA precursors appears to be less sensitive to a larger window size than non-miRNA structures.

### Classification of stem-loop structures

The motif-based genetic programs (see Methods) act as predictors that return a binary decision about whether an input sequence/structure contains a potential pre-miRNA or not. Predictions from multiple classifiers (16 here) are combined by voting with threshold, i.e., the minimum number of individual decisions required for an overall positive decision. Higher thresholds yield fewer structures predicted as miRNAs, but with a higher confidence.

**Figure 3:**
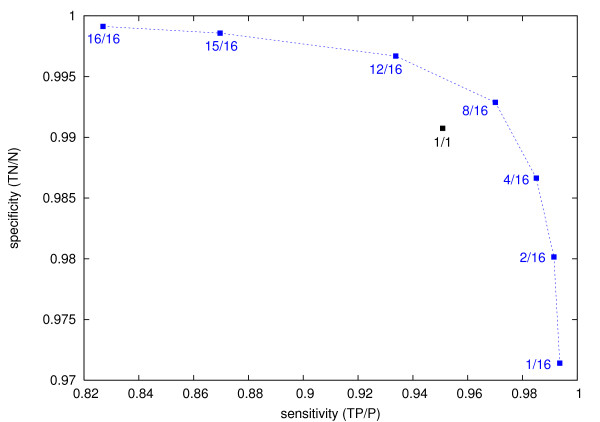
Performance of the ensemble classifier including 16 individual classifiers for different voting thresholds, i.e., minimum numbers of required positive decisions. The maximum threshold (16/16) achieves a specificity of above 99.9 percent on an independent test set of randomly selected sequences while still maintaining a sensitivity of above 82 percent on all human miRNAs (also used for training). Majority voting (8/16) shows more balanced values, both higher than found for the individual classifiers (1/1).

The ROC curve in Figure 3 shows the performance of the ensemble classifier for different voting thresholds. Values are reported for an independent negative test set of 100,000 windowed sequences (different from the training set) and for all positive examples (as used for training). Majority voting (8/16) shows rather balanced prediction accuracies on the negative (99.3 percent) and positive (97 percent) examples. Such a configuration is appropriate if one wants to decide on certain candidate sequences as, e.g., suggested by other methods.

When searching wider genome regions for new miRNAs, however, a higher specificity is desirable to keep the hit rate and number of potentially false positives low. Voting with maximum threshold (16/16) requires that all individual classifiers must vote "yes" and achieves a specificity of above 99.9 percent. This results in 87 (140) positive predictions out of 100,000 negative structures, excluding (including) multiple matches of directly successive windows. The small increase in specificity is paid by a more significant drop in sensitivity to around 82 percent here. A certain amount of missed true positives is acceptable, if new miRNAs are predicted with a higher confidence instead.

The performance of the individual classifiers (1/1) is at around 99.1 percent specificity and 95 percent sensitivity. However, this also means an almost 10 times higher rate of (potentially false) positive predictions.

### Cross-validation test

In a separate experiment five-fold cross-validation was applied to verify the performance of the classifier programs. Because GP is a probabilistic method, 5 independent GP runs were performed for each of the 5 positive training sets and the best program on each set was selected. The negative training sets were selected as described in Methods. On average, sensitivity remains at 95 percent over all human miRNAs and drops down to 90 percent on the respective set of miRNAs left out for testing. Specificity remains at an average of 99.1 percent over the 100,000 negative test examples.

Both values – specificity and sensitivity – compare well to what has been previously reported for other comparable *ab initio *methods, including both pioneering approaches [15,16] and second generation predictors [17,18,27,28]. Two very recent articles, officially published after the submission of our paper, report high performance values for feature-based classifiers. Kwang Loong and Mishra [27] achieve around 98 percent specificity at 84.5 percent sensitivity on human miRNAs. Their method applies a SVM with Gaussian radial basis function (RBF) kernels and an improved extraction of structural and sequential features. Jiang *et al. *[28] report even 95 percent sensitivity with about the same specificity (98 percent), found, however, by resampling over a training set with relatively few positive and negative examples (around 160 each [15]). These values reduce to 89 sensitivity and 93 percent specificity when testing the method on an independent set of positive and negative examples. Direct comparisons with performance values from the literature must be taken carefully, of course, due to different information, data sets, and prefilters used. Also note that we favor a specificity-sensitivity tradeoff towards higher specificity in this paper.

MiRNAs of the same family are given names with the same (integer) number but different types of suffixes that relate to the similarity of the mature sequence [21]. Numeric suffixes (-1,-2,...) indicate that the mature sequences are identical. Lettered suffixes (a,b,...) indicate that there are few mutations in the mature part. To exclude the influence of same-family members on the cross-validation results, we (1) remove all human miRNAs from each left-out test set, which have a member of the same family in the respective training set, and (2) keep only one member of each family in a test set. This reduces the average test set size to 62 (from 94 before). The average sensitivity is affected only slightly and is still around 87 percent (compared to 90 percent before).

### Performance on other miRNA data sets

To validate our ensemble classifier on different sets of positive examples, we extracted fixed-length windows centered around known miRNA sequences in mouse and rat, as described above for human.

There are 363 (228) mouse (rat) miRNA hairpins reported in miRBase 9.0 – with known genome location and without double sequences. We exclude 54 (42) which have an identical precursor in human. These have been found by matching the central 70 nt – the approximate precursor sequence – against the full human windows.

Our method correctly predicts 74 (81) percent of all miRNAs in mouse (rat) with the highest confidence, i.e., 100 percent agreement between all classifiers. If minimum 50 percent agreement is required for a positive decision the classification rate increases to 91 (98) percent. Only 97 (22) mouse (rat) miRNAs have no member of the same family in human, due to the high sequence conservation of (known) miRNAs. 71 (76) percent of these are found with maximum voting threshold and 87 (100) percent with majority voting. This also demonstrates that our classifiers while trained on human data are able to generalize to miRNAs of other species.

In addition, we test the performance of our combined classifier on 60 new human miRNA sequences in miRBase 10.0 of which 44 are founding members of new miRNA families. From the first set, 73 percent are found with maximum confidence and 90 percent with confidence ≥50 percent. For the second set, these numbers drop to 68 and 86, respectively.

Comparing the new structure sequences by their string edit distance [29] reveals that some are quite different to any previously known human precursor structure or belong to previously rather underrepresented structure classes (in miRBase 9.0 and our training set) with, e.g., larger asymmetric bulges in the stem arm.

### MicroRNA identification during genome scanning

Most miRNA genes are located in DNA regions which previously have been considered as non-coding regions, including intergenic regions and introns. Especially intronic miRNAs seem to be much more frequent than previously thought [30]. Many miRNAs appear in clusters on the genome, either in introns of a host gene or in polycistronic transcripts [31,32]. Such clusters are likely transcribed together and usually control mRNAs of related functions. Therefore, genomic regions around known miRNA locations are particularly promising for finding new miRNAs [12,16].

As a more realistic test scenario, we scan regions on chromosome 19 which has the highest number of validated miRNAs among all human chromosomes. The 66 miRNAs are grouped into 16 clusters with a minimum distance of 20 kilobases. The 100 nt wide scanning window is moved in 5 nt steps, starting 10 kb upstream of each cluster and ending 10 kb downstream.

The prefilter already identifies 56,142 (63 percent) from the total 88,808 structure sequences as negatives. The combined classifier finds 295 positive predictions (with 100 percent agreement) which is about twice as many as for random locations used above, indicating a higher number of true positive miRNAs. After removing double matches by overlapping windows these reduce to 173, of which 56 are rediscovered known miRNAs (85 percent of 66). The remaining 10 were not filtered out beforehand and all lie on the sense strand (which is scanned). This leaves 117 candidates as potentially new miRNAs.

We can use the results for the random locations – which are more likely true negatives – to estimate the number of real miRNAs out of all positive matches here. Of the 173 hits approximately 87** × **88, 808/100, 000 = 77 are likely false positives, while the remaining 173 **-** 77 = 96 are potentially real. Excluding the found known miRNAs leaves around 40 to be true positives.

Because it would be counterintuitive to scan regions around known miRNAs without using their information for finding new ones, miRNAs on chromosome 19 are part of the training set here. For comparison reasons, we trained another ensemble classifier without using these 66 miRNAs. Its sensitivity is only a little lower on chromosome 19 (52/66) and slightly better on all human miRNAs. One has to note here that neither the composition nor the size of the ensembles are particularly optimized.

### Postfiltering and analysis of miRNA candidates

Additional criteria may be applied on the positive predictions to obtain less miRNA candidates, that are more likely true. We focus on structural aspects again, including the free energy of matching structures and the number of directly successive matches of the scanning window.

**Figure 4:**
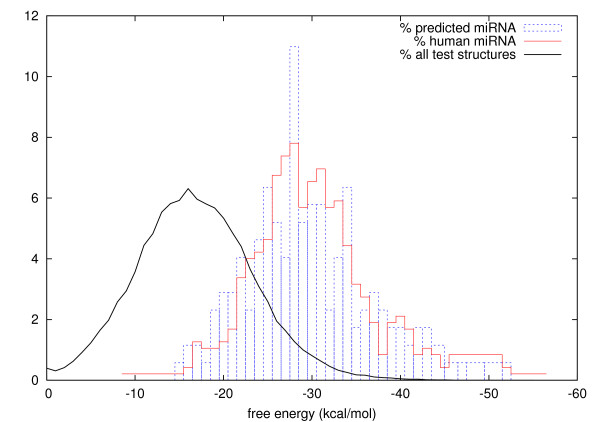
Free energy distributions of sequence windows (central 70 nt folding). Frequency distribution (in percent) of 173 predicted miRNAs on chromosome 19 matches the distribution of all 474 known human miRNAs (miRBase 9.0). The normal distribution of all tested 88,808 structures is significantly shifted towards higher free energies with mean around -15 kcal/mol, compared to about -30 for miRNAs. Structures with lower free energy, especially below -30, are more likely miRNAs. Energies are rounded to integers.

MiRNA structures are known to have lower free energy, i.e., to be more stable, than other non-coding or random structures [33]. Figure 4 compares different frequency distributions over free energies. The distribution of all 173 predicted miRNAs on chromosome 19 nicely fits the distribution of all 474 known human miRNAs. Both distributions are shifted by around -15 kcal/mol towards lower free energies, compared to the distribution of all 88,808 structures tested. So at least according to their free energy, most candidate structures seem to be real miRNAs, including especially those with free energy lower than -30. All energies relate to the folding of only the central 70 nt in each sequence windows. This is also why the free energy of 6 human miRNAs falls below -15 kcal/mol.

**Figure 5:**
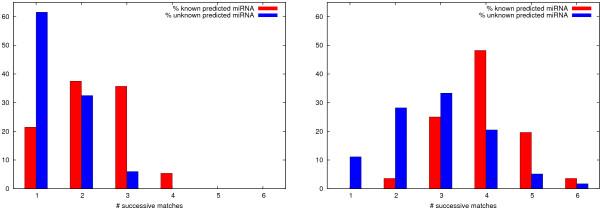
(*left*) Frequency distribution (in percent) over the number of directly successive sequence windows whose corresponding structure is a full match, i.e., is predicted positive by all 16 classifiers. Higher number of matches for known miRNAs indicates higher stability of folding. (*right*) Distribution counting also partial matches with the proportion of positive predictions being < 1 and within a range of three window shifts before and after a full match. Matches are averaged and rounded to integers.

The number of directly successive sequence windows is measured in two different ways. Figure 5(*left*) shows the frequency distribution over the number of successive matches by the sequence window, separately for the 117 unknown hits and the 56 known hits. Only full matches are counted, meaning that the corresponding structure must be predicted positively by all 16 classifiers. Known (true) miRNA locations match more frequently during scanning than unknown locations (probably not all true). More than 60 percent of the unknown candidates match only once, while this is true for only around 20 percent of the known candidates. On the other hand, around 40 percent known ones, but only 5 percent unknown ones match three times or more. This shows that miRNA structures are more independent from their position in the scanning window than other structures and is another indicator for their above-average stability, besides a lower free energy.

Here we only note that we found similar distributions of successive matches (1) over the 52 miRNAs redetected by the ensemble not trained on miRNAs from chromosome 19 and (2) when scanning over the 60 new miRNAs in miRBase 10.0 (see Section *Performance on other miRNA data sets*). That is, clearly more miRNAs are predicted with at least three matches than with only one match.

A more detailed picture is given in Figure 5(*right*) where the distributions also include partial matches by any number of classifiers. Depending on the proportion of positive predictions, full matches count 1 and partial matches < 1. These values are summed up from three window shifts before to three window shifts after a full match or a series of full matches. According to this distribution, 40 percent of the unknown predictions fall below 3 counts, but less than 5 percent of the known ones do.

**Figure 6:**
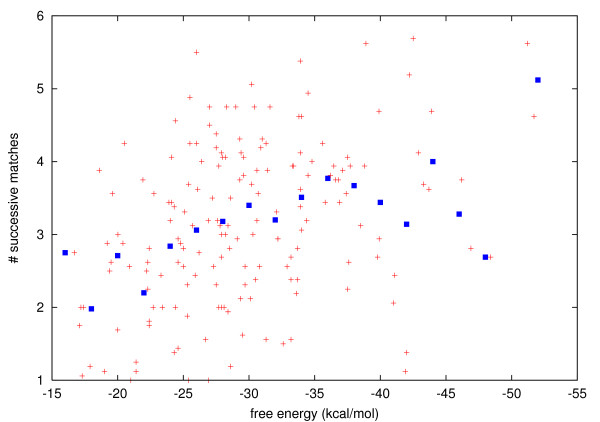
Free energy plotted against number of successive matches by the scanning window. Means over energy bins (highlighted in blue) reveal weak, but pronounced correlation between lower free energy and higher number of matches. Preceding and succeeding partial matches are included.

How similar are the different measures of structural stability? Figure 6 reveals a weak, but pronounced correlation between higher number of successive matches and lower free energy of the first matching window. This implies that the measures still capture sufficiently different aspects and are not interchangeable. Also note that the free energy alone is not an absolutely reliable indicator of structural stability.

One may derive different thresholds from these distributions to further select the 117 unknown predicted structures. For instance, a higher maximum free energy threshold of -27 would leave only 71 candidates. When applying an additional minimum threshold of 3 on the number of matches (second measure) the number of candidates reduces to 47. This is about the number of true positives estimated in the previous section. Also note that both thresholds are still passed by the vast majority (45 from 56) of known predicted miRNAs on chromosome 19.

### Transcription

A necessary precondition for a miRNA candidate to be true is that its genomic location is transcribed. A large number of transcripts is known not to be translated into proteins, but not all of these are necessarily functional. Non-coding RNAs not only occur between (protein-)coding genes, but often overlap with coding regions on the same or the opposite strand [34].

Alignment of nucleotide sequences to an EST library offers one way to identify coding and non-coding genes. *Expressed Sequence Tags *(ESTs) are relatively short (about 300–600 nt) and inaccurate (around 2 percent errors) subsequences of transcribed and spliced cDNA, synthesized from mature mRNA. More than 8 million human ESTs are now available in the GenBank dbEST database (release 062207) [35]. This library favors the sampling and, thus, the detection of rare and lowly expressed transcripts.

We use BLAST [36] with standard settings to search for the 117 predicted miRNA sequences of 100 nt in the dbEST database. About 40 percent (48/117) match human ESTs with at least 95 percent sequence identity over a minimum alignment length of 80 nt. This compares to around 30 percent of all known human miRNAs (139/474), a value similar enough to provide additional confidence in the correctness of our predictions.

To find out how many candidate sequences lie in introns, we first align all sequence regions that have been scanned on human chromosome 19 (see Section *MicroRNA identification during genome scanning*) to the EST library. If an EST alignment is split up into several discontiguous segments, we extract the whole sequence between the two outermost alignments, provided that these are longer than 60 nt. Then the intermediate regions are most likely introns which have been spliced out. A miRNA candidate is identified as being intronic, if it aligns to an extended EST, but not to the original (spliced) EST. In total we found 35 percent (41/117) of the candidates to match EST-defined introns and more than 75 percent ((48 + 41) / 117) to be transcribed. By comparison, only 12 out of the 66 known miRNAs on chromosome 19 match ESTs while 44 match introns.

### Knowledge extraction

Unlike black-box classifiers, like SVMs, our GP solutions may be better interpreted and analyzed in terms of the prediction model and the rules learned. In particular, the contained regular expressions provide an insight into what sets miRNAs apart from other structures.

A simple way of interpretation is to extract all structure motifs used in programs and match these separately against all positive and all negative examples. General rules about how miRNA structures should look like or not may be derived from highly distinctive expressions which match either significantly more miRNA or non-miRNA structures. In doing so, one has to keep in mind that the expressions were embedded in a more complex classifier structure and used in combination with other REs for the final decision making. For instance, motifs with a positive matching rate above 50 percent also match at least 10 percent of the negative examples. A single motif (as defined in Methods) is not enough to capture all information held in a miRNA precursor and matches the structure sequence only in parts (partial motifs). 

**Figure 7:**
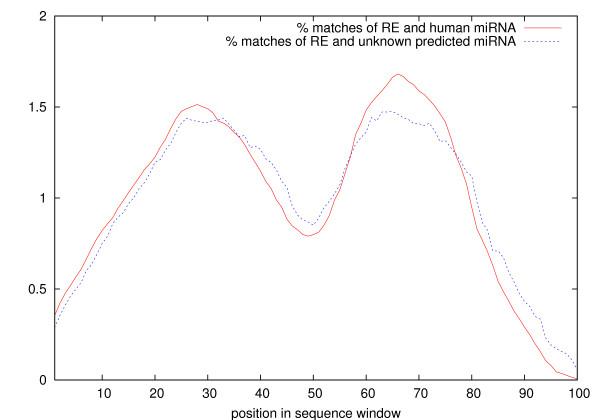
Frequency distribution (in percent) of expression-sequence matches over the window positions. 100 nt windows centered around all 474 known human miRNAs and all 117 unknown predicted miRNAs. Only regular expressions that match mostly positive structures are used. Loop region (approximately central) and flanking regions are less matched than stem sequences.

Figure 7 shows frequency distributions of expression-sequence matches over the 100 window positions. 33 "positive" regular expressions have been matched against the secondary structure of sequence windows centered around (1) all known human miRNAs and (2) all new miRNA candidates predicted here. All selected expressions have an at least three times higher matching rate on the positive structures than on the negative structures. The average length of a matching subsequence is around 30 nt (including wildcard matches, see Methods). The close similarity of both distributions gives at least some additional support for the similarity of the matched structures.

The terminal loop region (approximately in the window center) is matched clearly less frequently than the stem regions. In fact, only few expressions match over the full hairpin loop and even fewer contain subexpressions which determine loop sizes precisely. The matching frequency is even lower for the flanking sequences (~15 nt from each window end). Apparently, these regions are at least less important for finding characteristic structure motifs. This result is in line with the most distinctive features of miRNA precursors and predicted non-miRNA structures derived from secondary structure clustering in [37], which are size and number of bulges rather than the terminal loop size.

One explanation may be found in the conservation profile over miRNA precursor sequences which is saddle-like [12,13] and resembles the distribution graph in Figure 7. The higher variation in loop and flanking sequences also applies, to a certain extent, to the precursor structure [26]. Thermodynamic stability profiling reveals that the structure is apparently less robust in these regions [25,38]. Another reason may be that the secondary structure is predicted less accurately for the terminal loop than for the stem [38]. At least the range of predicted loop lengths may be too wide and variable to be used for a reliable identification of miRNAs.

## Conclusion

We introduced a new *ab initio *method for miRNA discovery in human that is purely based on finding distinctive motifs in predicted RNA secondary structure. In doing so, we favored a specificity-sensitivity tradeoff that is shifted towards higher specificity. Combining the predictions from multiple classifiers by voting with maximum threshold (100 percent agreement on a positive decision) has turned out to be a simple but effective means to increase confidence in and reduce the number of positive predictions and potentially false positives. 99.9 percent specificity requires an ensemble of 16 classifiers here. Sensitivity is still maintained at a level sufficient for genome scanning applications.

Another means to increase confidence is to postfilter the predicted miRNA candidates by imposing additional criteria. The robustness of the secondary structure against the position of the scanning window has been proposed as a structural criterion for miRNA identification. The sliding sequence window is more frequently identified as a match over a miRNA location than over a non-miRNA location.

Finally, we collected different statistical and empirical evidence to validate the correctness of our predictions. All 117 miRNA candidates structures match the free energy distribution of known miRNA precursors that is significantly shifted towards lower free energies, compared to the distribution of non-miRNA structures. Approximately one third was estimated to be true positives, i.e., real miRNAs or other small RNAs of similar structure. By aligning the candidate sequences to a human EST database, we found that above 75 percent are most likely transcribed and that around 35 percent are located in introns. 

The performance of our motif-based GP approach has been found at least competitive to presently existing methods for *ab initio *miRNA identification. More general advantages over SVM-based methods include:

(1) Less assumptions are made about how a pre-miRNA structure has to look like. The patterns (motifs) used for classification are not predefined, but are automatically derived from positive and negative examples during training (see Methods). SVMs, instead, requires certain "rules" to be set up which determine how a structure sequence is transformed into a feature vector.

(2) A preselection of candidate hairpins is not required since regular expressions are relatively independent from the absolute position of matching substructures. SVM classification, instead, requires hairpin-like structures to measure certain quality features.

(3) Because GP is a probabilistic method, a gain in performance is possible by combining multiple classifiers even if these have been trained on the same data. Deterministic SVMs only produce a single solution in this case.

(4) Our motif-based programs give a more direct and unbiased insight into what rules have been learned. Mostly the stem region of hairpin structures is used for miRNA prediction, in contrast to loop region and flanking sequences. A more detailed analysis of programs and structure motifs is a subject of our ongoing research.

As indicated above, *ab initio *miRNA finding, by definition, does not apply homology search or conservation. For instance, we did not prefilter the input sequences by scanning only regions conserved between human and, e.g., mouse or rat. Only 4 (3) of the potential miRNA sequences found in human are highly conserved in mouse (rat), i.e., with above 90 percent sequence identity over an alignment of at least 80 nt.

In general, it may be doubted whether one computational method alone is sufficient to estimate reliably how many miRNAs actually exist in the human genome. 99.9 percent specificity is, of course, not the end of the story. The number of predictions produced is still large when scanning the complete genome and it may be questioned if these can be all real miRNAs. To find the true positives may still require additional support by other computational and/or experimental approaches, testing other criteria than the precursor structure. Nevertheless, identification methods like the one presented here serve as an important step toward filtering potential pre-miRNA candidates.

## Methods

### Motif-based linear genetic programming

MiRPred applies a regular expression-based variant of linear genetic programming (LGP), a machine learning technique that automatically develops imperative computer programs in an artificial evolutionary process [19]. Multiple regular expressions like, e.g., ({3,12}.{1,15}-{6,7}){5,18}, are coevolved with and combined in each program. These relatively simple motifs are found *de novo *and are built from maximum 6 characters including parentheses and dots. Each character is followed by a {*min*, *max*} quantifier which specifies its allowed number in a structure sequence presented in dot-bracket notation (see Figure 1). Wildcard symbol "-" matches any character and allows distances (gaps) between subexpression matches. All motifs in a program are first matched against the input sequence. Then the actual classification rules are applied to these matching results, i.e., numbers of non-overlapping matches (mostly 0 or 1). Thus, a classifier consists of two parts, a matching part and a calculation part (see example in Additional file 1). The final continuous program output is mapped to a Boolean value (yes/no decision) depending on whether it is closer to 1 or -1. A small maximum number of 20 motifs per program compared to a relatively large number of examples makes overtraining (overspecialization) at least difficult.

### Training issues

Already the probabilistic nature of GP makes that two training runs can produce different results (programs). In order to make the combination of multiple programs more efficient, we use a different negative training set in each run including around 10 times as many structures as there are positive examples (468 human miRNA structures after prefiltering). Such a relation reflects very roughly the distribution in the genome and the much higher variety of non-miRNA structures.

Prediction performance is calculated during training as the mean of positive classification error rate and negative classification error rate:

12⋅((1−TPP)+(1−TNN))
 MathType@MTEF@5@5@+=feaagaart1ev2aaatCvAUfKttLearuWrP9MDH5MBPbIqV92AaeXatLxBI9gBaebbnrfifHhDYfgasaacPC6xNi=xI8qiVKYPFjYdHaVhbbf9v8qqaqFr0xc9vqFj0dXdbba91qpepeI8k8fiI+fsY=rqGqVepae9pg0db9vqaiVgFr0xfr=xfr=xc9adbaqaaeGacaGaaiaabeqaaeqabiWaaaGcbaqcfa4aaSaaaeaacqaIXaqmaeaacqaIYaGmaaGccqGHflY1daqadaqaamaabmaabaGaeGymaeJaeyOeI0scfa4aaSaaaeaacqWGubavcqWGqbauaeaacqWGqbauaaaakiaawIcacaGLPaaacqGHRaWkdaqadaqaaiabigdaXiabgkHiTKqbaoaalaaabaGaemivaqLaemOta4eabaGaemOta4eaaaGccaGLOaGaayzkaaaacaGLOaGaayzkaaaaaa@427A@

to balance the influence of the differently sized sets. *P *(*N*) denotes the total number of positive (negative) examples, while *TP *(*TN*) is the number of true positive (true negative) predictions.

## Authors' contributions

MB designed the method, carried out the experiments, and wrote the manuscript. CW participated in the design of the study and in the statistical analysis. Both authors read and approved the final manuscript.

## Supplementary Material

Additional file 1 Example of an individual classifier program (in C notation). Each program consists of a matching part with regular expressions and a calculation part. Continuous output is mapped to Boolean value (yes/no decision). Unused regular expressions and code lines not shown. Click here for file
